# Effect of local cold application during exercise on gene expression related to mitochondrial homeostasis

**DOI:** 10.1139/apnm-2020-0387

**Published:** 2020-09-22

**Authors:** Ben Meister, Chris Collins, Mark McGlynn, Dustin Slivka

**Affiliations:** School of Health and Kinesiology, University of Nebraska at Omaha, Omaha, NE 68182, USA.

**Keywords:** cycling, mRNA, skeletal muscle, PGC-1α, endurance exercise, temperature, mitochondria, cyclisme, ARNm, muscle squelettique, PGC-1α, exercice d’endurance, température, mitochondries

## Abstract

Exercise training increases mitochondrial content in active skeletal muscle. Previous work suggests that mitochondrial-related genes respond favorably to exercise in cold environments. However, the impact of localized tissue cooling is unknown. The purpose of this study was to determine the impact of local muscle cooling during endurance exercise on human skeletal muscle mitochondrial-related gene expression. Twelve subjects (age, 28 ± 6 years) cycled at 65% peak power output. One leg was cooled (C) for 30 min before and during exercise with a thermal wrap while the other leg was wrapped but not cooled, room temperature (RT). Muscle biopsies were taken from each vastus lateralis before and 4 h after exercise for the analysis of gene expression. Muscle temperature was lower in the C (29.2 ± 0.7 °C) than the RT (34.1 ± 0.3 °C) condition after pre-cooling for 30 min before exercise (*p* < 0.001) and remained lower after exercise in the C (36.9 ± 0.5) than the RT (38.4 ± 0.2, *p* < 0.001) condition. *PGC-1α* and *NRF1* mRNA expression were lower in the C (*p* = 0.012 and *p* = 0.045, respectively) than the RT condition at 4 h after exercise. There were no temperature-related differences in other genes (*p* > 0.05). These data suggest that local cooling has an inhibitory effect on exercise-induced *PGC-1α* and *NRF1* expression in human skeletal muscle. Those considering using local cooling during exercise should consider other systemic cooling options.

**Novelty:**

- Local cooling has an inhibitory effect on exercise-induced *PGC-1α* and *NRF1* expression in human skeletal muscle.
- Local cooling may lead to a less robust exercise stimulus compared with standard conditions.

## Résumé

L’entraînement physique augmente le contenu mitochondrial du muscle squelettique actif. Des travaux antérieurs suggèrent que les gènes liés aux mitochondries répondent favorablement à l’exercice dans des environnements froids. Cependant, on ne connait pas l’impact du refroidissement localisé des tissus. Le but de cette étude est de déterminer l’impact du refroidissement musculaire local lors d’un exercice d’endurance sur l’expression génique liée aux mitochondries du muscle squelettique humain. Douze sujets âgés de 28 ans ± 6 ans pédalent à 65 % de puissance maximale. Une jambe est refroidie (« C ») pendant 30 minutes avant et pendant l’exercice avec une enveloppe thermique tandis que l’autre jambe est enveloppée mais non refroidie pour rester à la température ambiante (« RT »). Pour l’analyse de l’expression génique, des biopsies musculaires sont prélevées sur chaque *vastus lateralis* avant et 4 heurès apres l’exercice. La température musculaire est plus basse dans la condition C (29,2 ± 0,7 °C) qu’en RT (34,1 ± 0,3 °C) après le prérefroidissement de 30 minutes avant l’exercice (*p* < 0,001) et reste plus basse après l’exercice dans la condition C (36,9 ± 0,5) qu’en RT (38,4 ± 0,2, *p* < 0,001). L’expression de l’ARNm de *PGC-1α* et *NRF1* est plus faible dans la condition C (*p* = 0,012 et *p* = 0,045, respectivement) qu’en RT 4 h après l’exercice. Il n’y a pas de différences liées à la température dans d’autres gènes (*p* > 0,05). Ces données suggèrent que le refroidissement local a dans le muscle squelettique humain un effet inhibiteur sur l’expression de *PGC-1α* et *NRF1* induite par l’exercice. Les personnes qui envisagent d’utiliser le refroidissement local pendant l’exercice devraient envisager d’autres options de refroidissement systémique. [Traduit par la Rédaction]

**Les nouveautés:**

- Le refroidissement local a dans le muscle squelettique humain un effet inhibiteur sur l’expression de *PGC-1α* et *NRF1* induite par l’exercice.
- Le refroidissement local peut conduire à un stimulus d’exercice moins intense par rapport aux conditions standard.

## Introduction

Mitochondrial dysfunction is believed to play a role in many vascular diseases (Makris et al. 2007; Bullon et al. 2014), aging (Derbré et al. 2012), obesity (Bournat and Brown 2010), metabolic diseases (Bullon et al. 2014), and inflammatory and neurodegenerative diseases (Witte et al 2010). Endurance exercise training can increase mitochondrial content in active skeletal muscle (Hoppeler et al. 1973). Many individuals with mitochondrial-related pathologies have limited capacity to exercise, which highlights the need to enhance the effectiveness of a single exercise bout. Consequently, it is important to evaluate novel interventions that may accelerate mitochondrial development for therapeutic use in these special populations.

Healthy mitochondrial function is dependent on mitochondrial homeostasis, which comprises a balance of mitochondrial growth (biogenesis) and mitochondrial breakdown (mitophagy). Research from animal models suggest that cold temperatures are associated with enhanced mitochondrial biogenesis in skeletal muscle (Puigserver et al. 1998; Wu et al. 1999; Oliveira et al. 2004). In mice, chronic exercise is a prerequisite for increases in peroxisome proliferator-activated receptor gamma 1-alpha (*PGC-1α*) expression in response to cold (Seebacher and Glanville 2010). Similarly, humans exposed to 7 °C temperature for 3 h without exercise show no alterations in mitochondrial-related gene expression (Zak et al. 2017). However, when exercise is coupled with cold exposure, an enhanced exercise-induced *PGC-1α* messenger RNA (mRNA) response is observed (Slivka et al. 2012, 2013). Thus, exercise appears to be a critical component for cold to enhance mitochondrial adaptation. The exact mechanistic stimulus for the cold-induced exercise response is currently unknown. It may be related to core temperature, skin temperature, intramuscular temperature, or any combination of factors. By preventing an increase in core temperature during exercise in a rat model mitochondrial biogenesis in the muscle may be enhanced (Mitchell et al. 2002). In a human model, endurance exercise in the cold (7 °C) demonstrated higher *PGC-1a* mRNA than at room temperature, despite no differences in core temperature (Slivka et al. 2013). Thus, skin or intramuscular temperature alterations may be a stimulus that impacts the acute exercise response in human skeletal muscle.

Preliminary research utilizing cold application during recovery from exercise did not alter mitochondrial-related gene expression (Shute et al. 2017). It is possible that the extremely low intramuscular temperature in this study (27 °C) may have been too cold for optimal gene expression since many metabolic processes are slower in response to local cooling (Nemet et al. 2009), including glycogen synthesis (Slivka et al. 2012). These investigations established the recovery response of cold muscle temperature but do not directly address the response during exercise when muscle temperatures are increased. It is uncertain how human skeletal muscle responds to localized cold during endurance exercise. The purpose of this study was to determine the impact of local muscle cooling during endurance exercise on human skeletal muscle gene expression related to mitochondrial homeostasis.

## Materials and methods

### Participants

Eleven recreationally active and apparently healthy participants (9 males and 2 females; age, 28 ± 6 years; weight, 81.8 ± 10.9 kg; 23.9% ± 6.1% body fat; maximal aerobic capacity ($\dot{V}O_{2\text{peak}}$) 42.9 ± 9.8 mL/(kg·min); peak power output (*W*_max_) 235 ± 48 W) visited the Exercise Physiology Lab on 2 occasions. Participants were informed of all procedures and risks before signing a University of Nebraska Medical Center Institutional Review Board approved consent document that conformed to the standards set by the *Declaration of Helsinki.* To be considered apparently healthy, subjects needed to be free from any signs or symptoms of health conditions that would impact their ability to complete the protocol. Recreationally active was defined as engaging in regular exercise twice per week for at least the last 8 weeks.

### Initial visit

Body fat was assessed through hydrostatic weighing using an electronic load cell-based system (Exertech, Dresbach, Minn., USA), correcting for estimated residual lung volume. A $\dot{V}O_{2\text{peak}}$ was conducted on an electronically braked cycle ergometer (Excalibur Sport, Lode, Groningen, Netherlands) using a graded-exercise protocol until volitional fatigue. Subjects began cycling at 95 W and the workload increased by 35 W every 3 min until volitional fatigue. The highest oxygen consumption recorded by a Parvo Medics TrueOne 2400 Metabolic Measurement System (Sandy, Utah, USA) was defined as the $\dot{V}O_{2\text{peak}}$. Maximal workload was calculated as the highest completed stage (in watts) + the proportion of time in the last stage multiplied by the 35 W per stage increment. For example, if the subject stopped 30 s into the 235 W stage, the highest completed stage would be 200 W because each stage the workload increases by 35 W. The maximal workload would be 200 W + (35 W × 30 s /180 s) because the subject completed 30 s of the 180-s stage, which would equal 205 W. The workload for the experimental trial was set at 65% of the maximal workload.

### Experimental trial

The initial visit and the experimental trial were separated by at least 48 h to allow for adequate recovery. Participants were instructed to eat a light meal 30 min before arriving. In addition, participants were asked to avoid consuming alcohol, tobacco, or other drugs for at least 20 h before the experimental visit. On the trial day, subjects arrived in the morning having refrained from exercise within the previous 24 h. An initial muscle biopsy, muscle temperature, and skin temperature was obtained after 30 min of supine rest to represent the resting (no temperature or exercise intervention) control condition for each leg (Pre).

After the initial biopsy and temperature measurements, thermal wraps (ThermaZone; Innovative Medical Equipment LLC, Cleveland, Ohio, USA) that circulated fluid were positioned around each thigh for 30 min prior to exercise and worn throughout the exercise session. The fluid circulating through the wrap was maintained at 0 °C using a computer-controlled cooling device (Zamar Cube; Zamar Medical, Poreč, Croatia). One leg was cooled (C) by the thermal wrap, and the other leg served as the room temperature control (RT). The RT limb still had a wrap placed around the thigh to match any potential compression or insulation effects but did not have cold fluid circulating. The C leg and the RT leg were randomly assigned. After the 30-min precooling (Post-Cool), skin and muscle temperature were remeasured.

Next, participants cycled for 1 h at 65% of the power output associated with $\dot{V}O_{2\text{peak}}$, with the thermal wraps remaining in place around each leg. A 500-mL bottle of water was provided to each participant to drink ad libitum during exercise. Immediately following the exercise bout (99 ± 33 s), the thermal pads were removed and muscle and skin temperatures were measured (Post-Exercise). Participants recovered for 4 h in a room-temperature environment and were instructed to refrain from physical exertion and eat only the food that was provided. A small meal of commercially prepackaged food items (730 ± 121 kcal; 42% carbohydrate, 15% protein; and 43% fat) was provided between 30 min after exercise and 1 h after exercise to ensure they were in an adequate nutritional status for recovery. The final biopsy was collected 240 ± 4 min after the end of the exercise bout. After the biopsy, final muscle temperatures and skin temperatures were obtained from each leg (4-h Post-Exercise). The order of the testing was (*i*) muscle biopsies, (*ii*) intramuscular temperature, (*iii*) bandaging, and (*iv*) skin temperature.

### Exercise trial data

Rating of perceived exertion (RPE) of the total exercise bout was assessed in the last 5 min of the hour-long cycling bout using the Borg RPE scale (Borg 1983). Heart rate was monitored continuously during the cycling exercise using a GARMIN heart rate monitor (HRM Dual; Garmin International Inc., Garmin Ltd., Olathe, Kans., USA). Power was monitored continuously by a GARMIN Edge 520 and GARMIN Vector 3 pedals (Garmin International Inc., Garmin Ltd.). The pedals measured the right and left power independently to determine the relative amount of work done by each leg to ensure that the work completed between legs was equivalent.

### Biopsies

Subjects rested in a supine position for 30 min prior to each biopsy. The biopsy site (~10 cm proximal to the patella and ~5 cm lateral from the center of the thigh in the belly of the vastus lateralis (VL)) was numbed with ~3 mL of 1% lidocaine injected under the surface of the skin and surrounding muscle fascia. The site was sterilized with betadine and small incision was made. The second biopsy on each leg was obtained from the same incision but the needle was inserted at a slightly different angle to sample an area proximal to the first. The 4 total muscle biopsies (2 from each leg) were obtained from the VL muscle using a ProMag Ultra Biopsy Instrument and a 14-gauge ProMag Ultra Biopsy Needle (Argon Medical Devices Inc., Athens, Texas, USA) at a depth of ~25 mm below the surface of the skin. Biopsy samples were obtained from each leg at Pre and 4-h Post-Exercise. There was a delay of 3.26 ± 1.25 min between biopsies that allowed the investigator to move between legs. Pre-biopsies were collected from each leg, allowing each leg to serve as its own resting control. The 4-h Post-Exercise time point was chosen because this is within the time course of peak expression of our target genes (Leick et al. 2010; Yang et al. 2006). After removal of any excess blood, connective tissue, and fat, tissue samples (12.0 ± 3.3 mg) were immersed in 100 μL All-Protect (Qiagen, Hilden, North Rhine-Westphalia, Germany) and stored at 4 °C overnight and then at −30 °C for later analysis. The muscle biopsies were used to evaluate alterations in the expression of specific genes related to mitochondrial biogenesis and mitophagy.

### Intramuscular temperature

Immediately after the initial muscle biopsies, while the incision was still open, a hypodermic (~26 g) thermocouple (Physitemp; Physitemp Instruments LLC, Clifton, N.J., USA) was inserted ~2–3 cm into the muscle until the temperature reading stabilized. An ExTech 4-Channel Temperature Meter (Extech Instruments, Nashua, N.H., USA) was used to log temperature data. After the muscle temperature measurement, the area was cleaned using alcohol wipes, treated with antibiotic, and bandaged.

### Skin temperature

Skin temperature was measured using a surface thermistor (Physitemp, Physitemp LLC) on the surface of each VL. The thermistor was held on the surface of the skin, 2 cm proximal to the bandage placed over the incision for 60 s. The ExTech 4-Channel Temperature Meter (Extech Instruments, Nashua, N.H., USA) allowed for the skin temperature measurements to be obtained from each leg at the same time.

### mRNA analysis

Muscle mRNA of specific genes was determined using quantitative real-time reverse transcriptase polymerase chain reaction (qRT-PCR). Skeletal muscle samples (12.0 ± 3.3 mg) were homogenized in 800 μL of Trizol (Invitrogen, Carlsbad, Calif., USA) using an electric homogenizer (Tissue Tearor, Biosped Products Inc., Bartlesville, Okla., USA). Samples were incubated at room temperature for 5 min, 160 μL of chloroform per 800 μL of Trizol was added, and the tubes were shaken by hand for 15 s. After another short incubation at room temperature (2–3 min) the samples were centrifuged at 12 000*g* for 15 min. The aqueous phase was transferred to a fresh tube, 400 μL of isopropyl alcohol was added and incubated overnight at −20 °C to precipitate mRNA. The samples were then centrifuged at 12 000*g* for 10 min at 4 °C and the mRNA was washed by removing the supernatant and adding 800 μL of 75% ethanol. Samples were vortexed and then centrifuged at 7500*g* for 5 min at 4 °C. RNA was quantified (184.1 ± 78.4 ng/μL) with a nano-spectrophotometer (nano-drop ND-1000, Wilmington, De., USA).

Superscript-first-strand synthesis system for qRT-PCR (Superscript IV, Invitrogen) was used to convert RNA to complementary DNA (cDNA). Each sample within a subject was adjusted to contain a standard RNA concentration (3 ng/μL) by dilution using RNase free water. Each qRT-PCR 20-μL reaction volume contained 1 μL probe (5 μmol/L) and primer (10 μmol/L) mix (PrimeTime qRT-PCR assay, Integrated DNA technologies), 10 μL Brilliant III Ultra-Fast qRT-PCR master mix (Agilent Technologies Inc., Santa Clara, Calif., USA), 0.3 μL reference dye mixture, 4.2 μL deionized water, and 4.5 μL of sample cDNA. Samples were analyzed using an Agilent Technologies Aria Mx real time PCR detection system (Agilent Technologies Inc.) running 1 cycle at 95 °C for 3 min, then 40 cycles of 95 °C for 5 s, and 60 °C for 10 s.

Quantification of mRNA for genes of interest was calculated using the 2^−ΔΔCT^ method (Livak and Schmittgen 2001) relative to stable reference genes and to the pre-intervention time-point (1.0-fold change). The geometric mean of the following 3 reference genes were used as the stable reference point: beta-actin (*ACTB*), ribosomal protein S18 (*RPS18*), and glyceraldehyde-3 phosphate dehydrogenase (*GAPDH*) for each participant. NormFinder software (Andersen et al. 2004) was used to examine reference gene stability. The stability value of the geometric mean of the reference genes was 0.021. Additionally, a 2-way repeated-measures ANOVA revealed no differences in the expression of the geometric mean of the reference genes over time or between conditions (*p* > 0.05). Average coefficient of variation for the house-keeping gene triplicates was 0.36% ± 0.78%. Probes and Primers that targeted the specific gene sequences in Table 1 were attained from Integrated DNA Technologies (Coralville, Iowa, USA) as previously described (Opichka et al. 2019). The muscle samples were analyzed for the transcription of genes associated with mitophagy (PTEN-induced putative protein kinase 1 (*PINK1*), Parkin RBR E3 Ubiquitin Protein (*PARK2*), Bcl-2/adenovirus E1B 19-kDa interacting protein (*BNIP3*), and BNIP3-like (*BNIP3-L*). The following genes are associated with mitochondrial biogenesis: *PGC-1α*, transcription factor A mitochondrial (*TFAM*), estrogen-related receptor alpha (*ERRα*), nuclear respiratory factor 1 (*NRF1*), nuclear respiratory factor 2 (*NRF2*), and vascular endothelial growth factor (*VEGF*).

### Statistical analysis

A repeated-measures 2-way ANOVA (2 × 2; time × condition) was used for the statistical analysis of dependent variables. ΔΔCT values from mRNA analysis are not normally distributed; therefore, statistical analysis of mRNA was performed on log-transformed ΔΔCT data. If the *F* ratio was determined significant, then a Fisher’s protected Least Significant Difference post hoc was performed to evaluate where significance differences occurred. The probability of type I error of less than 5% was considered significant (*p* < 0.05). All statistical data were analyzed using the Statistical Package for Social Sciences software (version 25; IBM Corp., Armonk, N.Y., USA). Data are expressed as means ± SD unless stated otherwise.

## Results

### Exercise trial data

RPE during the 1-h cycling exercise bout was 16 ± 1. Maximal heart rate from the maximal oxygen uptake trial was 180 ± 12 bpm. Heart rate during the experimental session was 153 ± 15 bpm, which represents 85% ± 5% of the maximal heart rate from the oxygen uptake trial. Power was 153 ± 31 W. Power output was 51% ± 4% in the C leg compared with 49% ± 4% in the RT leg. Therefore, temperature had no effect on work performed by each limb (*p* > 0.05).

### Skin temperature

**Fig. 1.**
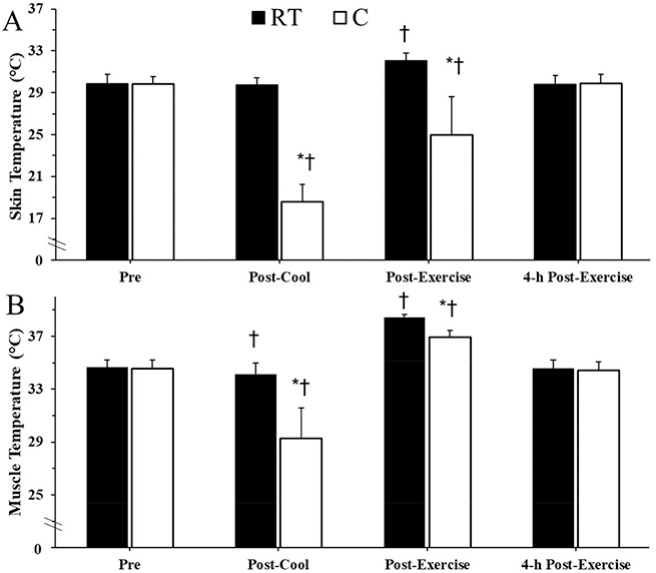
(A) Skin temperatures of each leg during the experimental trial. (B) Intramuscular temperatures of the vastus lateralis in each leg during the experimental trial. 4-h Post-Exercise, after the 4-h recovery; C, cooled; Pre, before the exercise session; Post-Cool, after 30-min pre-cooling; Post-Exercise, immediately following exercise; RT, room temperature control. Data are means ± SD. *, *p* < 0.05 from RT; †, *p* < 0.05 from Pre of the same limb.

Skin temperature was lower in the C than the RT condition at the Post-Cool time point (*p* < 0.001) and remained lower through Post-Exercise (*p* < 0.001). There were no differences in skin temperature between limbs at Pre (*p* = 0.972) or 4-h Post-Exercise (*p* = 0.803). Skin temperature decreased by 37% in the C (*p* < 0.001) but not in the RT (*p* = 0.687) condition from Pre to Post-Cool. Skin temperature increased by 7% in RT (*p* < 0.001) and by 34% in C (*p* < 0.001) from Post-Cool to Post-Exercise. Skin temperature in the RT condition at Post-Exercise was 7% higher than Pre (*p* < 0.001). Skin temperature in the C condition at Post-Exercise was still lower than Pre (*p* < 0.001). Skin tempera-ture returned to baseline by 4-h Post-Exercise (*p* > 0.05). See Fig. 1A.

### Intramuscular temperature

Intramuscular temperature was lower in the C than the RT condition at the Post-Cool time-point (*p* < 0.001) and remained lower at Post-Exercise (*p* < 0.001). Intramuscular temperature decreased by 15% in the C condition (*p* < 0.001) and by 1% in the RT condition (*p* = 0.01) from Pre to Post-Cool. Intramuscular temperature increased 26% from Post-Cool to Post-Exercise in the C condition (*p* < 0.001) and by 12% in the RT condition (*p* < 0.001). Muscle temperature returned to baseline by 4-h Post-Exercise (*p* > 0.05). There were no differences in muscle temperature between limbs at Pre or 4-h Post-Exercise (*p* > 0.05). See Fig. 1B.

### Gene expression

**Fig. 2.**
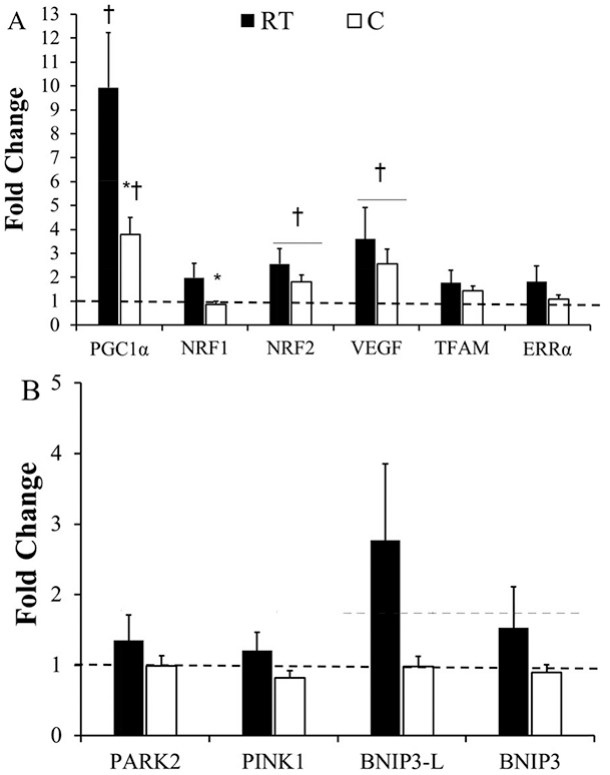
(A) Skeletal muscle mitochondrial biogenesis-related messenger RNA (mRNA) expression 4-h Post-Exercise. (B) Skeletal muscle mitophagy-related mRNA expression 4-h Post-Exercise.All 4-h Post-Exercise samples were normalized to Pre samples, represented as the dashed line at 1.0. 4-h Post-Exercise, after the 4-h recovery; C, cooled; Pre, before the exercise session; Post-Cool, after 30-min pre-cooling; Post-Exercise, immediately following exercise; RT, room temperature control. Data are means ± SEM. *, *p* < 0.05 from RT; †, *p* < 0.05 from Pre to 4-h Post-Exercise.

*NRF2* increased in both the C and RT conditions (*p* = 0.045, main effect) with exercise independent of temperature. *VEGF* mRNA increased in both the C and RT conditions (*p* = 0.008, main effect) with exercise independent of temperature. *PGC-1α* mRNA expression increased in both limbs (*p* < 0.001, main effect) but was lower in the C condition (*p* = 0.012, interaction) at 4-h Post-Exercise. Additional interactive effects were observed in a blunted *NRF1* mRNA expression in the C condition (C = 2.0 ± 0.6 vs. RT 0.9 ± 0.1-fold change; *p* = 0.045) at 4-h Post-Exercise. There were no differences in *NRF2*, *TFAM, VEGF,* or *ERRα* between temperature conditions (*p* > 0.05). There were no changes in *NRF1, TFAM,* or *ERRα* in response to exercise (*p* > 0.05). Gene expression data related to mitochondrial biogenesis are presented in Fig. 2A. There were no differences in *BNIP3, BNIP3-L, PINK1,* or *PARK2* between temperature conditions (*p* > 0.05) nor in response to exercise (*p* > 0.05). Gene expression data related to mitophagy are presented in Fig. 2B. Data are expressed as means ± SE.

## Discussion

The present study aimed to determine the potential impact of local muscle cooling during endurance exercise on *PGC-1α* and expression of other genes related to mitochondrial homeostasis. To our knowledge, this was the first study to examine both mitochondrial biogenesis and mitophagy mRNA responses to local muscle cooling during endurance exercise in humans. The main finding of this study is that the normal exercise-induced increase in *PGC-1α* is attenuated by local muscle cooling. This research may help guide development of temperature-optimized protocols to improve exercise outcomes in individuals with mitochondrial-related pathologies or those seeking increased athletic performance. This study provides the initial rationale for future investigation of the long-term adaptive response associated with altered muscle temperature. Specifically, local muscle cooling may lead to a less robust exercise stimulus when compared with standard conditions.

Environmental cold interventions can decrease whole-body temperature (Shute et al. 2018). Although we did not measure core temperature in this study, icing of the thigh area in our previous work (Shute et al. 2017) decreased muscle temperature in the VL to 26.7 °C without altering core temperature. In the current project, the thermal wraps decreased muscle temperature to an average of 29.3 °C. Therefore, with the relatively modest decline in muscle temperature it is unlikely that a sufficient stimulus was present to alter core temperature. As expected, local cold application before exercise only altered thigh skin and muscle temperature in the limb that received the treatment. Collectively, the results of this project, and that of our previous work (Shute et al. 2017), indicate that local cooling of a relatively small surface area only impacts the directly cooled area. During exercise, VL muscle temperatures increased above resting baseline in both legs. However, the muscle temperature was significantly higher in the RT compared with the C condition. Thus, we may consider the current cold application intervention to limit maximal heat in the exercising muscle. It is important to note that exposure to environmental cold is quite different than altering only small areas of local muscle and skin.

It is unclear if decreased transcription of *PGC-1α* and *NRF1* are a result of general alterations in transcription or a pathway dependent response to lower muscle temperature. Mitophagy targets dysfunctional mitochondria for degradation by modulating several genes including *PINK1, PARK2, BNIP3,* and *BNIP3-L.* Although not statistically significant, a similar pattern can be identified where expression was lower in the C than the RT condition (Fig. 2B). Collectively, the results of this investigation and that of our previous work (Opichka et al. 2019) suggest that the transcription of these mitophagy-related genes are not dependent on temperature.

This study was designed to provide insight on local muscle temperature. The thermal wraps utilized in this study were applied around the thighs to cool the VL, which is one of the primary muscles used during cycling exercise (da Silva et al. 2016). A lower muscle temperature during endurance exercise generally resulted in reduced transcription of genes related to mitochondrial homeostasis. Decreased muscle temperature has also been shown to decrease transcription of specific genes related to skeletal muscle growth (Zak et al. 2018), suggesting an overall reduction in transcription that may not be specific to any pathway. Warmer muscle temperatures may provide a more favorable metabolic environment (Webster 1987). Increased muscle temperature may be important for gene transcription in human skeletal muscle and lend to the effectiveness of exercise in general to increase transcription. Future research should assess the importance of increased muscle temperature for optimal transcription and desired responses.

Environmentally cold conditions applied during endurance exercise increases *PGC-1α* mRNA more than the same exercise at room temperature (Slivka et al. 2012, 2013). In the current project, *PGC-1α* mRNA increased with exercise but the exercise response was blunted when local cooling was applied. This indicates that different modes of cooling elicit different responses in *PGC-1α* expression. Altered muscle metabolism and substrate utilization may also provide an explanation for the differences in environmental and local cold-induced *PGC-1α* mRNA expression. The human physiological response to environmental cold is an increased metabolic rate (Slivka et al. 2012), with the majority of the increase coming from carbohydrate oxidation (Vallerand and Jacobs 1989). Whereas, the application of local cold reduces local blood flow, local metabolic rate, and glycogen synthesis (Tucker et al. 2012; Nemet et al. 2009). Exercise and a lower cellular energy state activates AMP-activated protein kinase (AMPK), Ca^2+^/calmodulin-dependent protein kinase IV (CaMKIV), calcineurin (CnA), and p38 mitogen-activated protein kinase (p38 MAPK), inducing *PGC-1α* transcription (Fernandez-Marcos and Auwerx 2011). AMPK regulates PGC-1α and are sensitive to changes in metabolism such as increased glucose utilization (Jäger et al. 2007). Thus, enhanced metabolic rate and carbohydrate oxidation with whole-body cold exposure could activate, while the lower local metabolism with local cooling may blunt this pathway and explain the differential *PGC-1α* response between environmental and local cold exposure. Local metabolic rate was not measured in the current project, but no shivering or whole-body thermal discomfort was observed, and both limbs were exposed to the same whole-body metabolic rate.

An additional stimulus that can regulate *PGC-1α* is cold stimulation of skin B_3_-adrenergic receptors (Fernandez-Marcos and Auwerx 2011). During environmental cold exposure a large area of skin receptors would be stimulated and could contribute to the observed elevation of *PGC-1α* expression (Slivka et al. 2013, 2012). During local cooling of a relatively small area, as in the current study, not as many skin receptors would be stimulated and thus not further stimulate *PGC-1α* expression. The skin thermal reception may not explain the observed reduced *PGC-1α* expression, but does contribute to the explanation of why *PGC-1α* expression was not increased in the cooled limb and further contributes to the notion that environmental cold and local cold are very different physiological stimuli.

Reduced muscle temperature decreases maximal dynamic strength and power output during cycling (Bergh and Ekblom 1979). Thus, differences between a cooled limb and control limb could be related to the relative workload of each limb during cycling. If the relative work of the cooled limb was reduced, we would expect the lower observed *PGC-1α* response. However, during our cycling protocol we did not observe differences in workload between the limbs and thus differences in workload do not likely explain the differences in *PGC-1α* expression.

Icing and local cold application is a common therapy for pain management and inflammation. Although exercise and an active lifestyle are the most prominent therapies to improve mitochondrial health, therapeutic modalities that amplify the benefits of a single exercise bout warrant further attention. None of our participants had difficulty completing the 1-h cycling exercise at 65% *W*_peak_ with the thermal wraps. However, this protocol may not be feasible for sedentary and diseased populations where mitochondrial dysfunction is implicated. Future clinical research may identify a temperature-optimized therapy for populations with limitations to exercise. Exercise in a cold environment appears to enhance the gene expression related to mitochondrial biogenesis compared with a room temperature environment (Puigserver et al. 1998; Slivka et al. 2012, 2013; Chung et al. 2017) and provide a more conducive transcriptional environment for mitochondrial development. The current data suggest that local cold does not illicit the same response as environmental cold when coupled with endurance exercise. In fact, local cold applied during exercise appears to reduce the gene expression related to mitochondrial biogenesis. Those considering using icing or local cooling during exercise for ergogenic benefits should consider other systemic cooling options.

## Figures and Tables

**Table 1.** Probes and primers used for real-time reverse transcription quantitative PCR.

	Primer 1	Primer 2	Probe
Reference			
ACTB	AAGTCAGTGTACAGGTAAGCC	GTCCCCCAACTTGAGATGTATG	CTGCCTCCACCCACTCCA
RPS18	GTCAATGTCTGCTTTCCTCAAC	GTTCCAGCATATTTTGCGAGT	TCTTCGGCCCACACCCTTAATGG
GAPDH	TGTAGTTGAGGTCAATGAAGGG	ACATCGCTCAGACACCATG	AAGGTCGGAGTCAACGGATTTGGTC
Mitophagy			
PINK1	GTTGCTTGGGACCTCTCTTG	TGAACACAATGAGCCAGGAG	TGTAAGTGACTGCTCCATACTCCCCA
PARK2	GCTTGGTGGTTTTCTTGATGG	TTGAAGCCTCAGGAACAACT	CCTGCTCGGCGGCTCTTTCA
BNIP3	CCACTAACGAACCAAGTCAGAC	CATCTCTGCTGCTCTCTCAT	AAAGGTGCTGGTGGAGGTTGTCA
BNIP3L	CAAACATGATCTGCCCATCTTC	TCCTCATCCTCCATCCACAA	TCTCACTGTGACAGCCCTTCGC
Biogenesis			
PGC-1α	GCAATCCGTCTTCATCCACA	CCAATCAGTACAACAATGAGCCT	AGCAGTCCTCACAGAGACACTAGACAG
NRF1	GTCATCTCACCTCCCTGTAAC	GATGCTTCAGAATTGCCAACC	ATGGAGAGGTGGAACAAAATTGGGC
NRF2	TGTAGTCTTGGTTCTAGCAGTTTC	TGGAACAGAGAAAGCAGAGTG	TGGTTCATTGAT GTCTATGGCCTGGC
TFAM	GCCAAGACAGATGAAAACCAC	TGGGAAGGTCTGGAGCA	CGCTCCCCCTTCAGTTTTGTGTATTT
ERRα	TCTCCGCTTGGTGATCTCA	CTATGGTGTGGCATCCTGTG	TGGTCCTCTTGAAGAAGGCTTTGCA
VEGF	GCGCTGATAGACATCCATGA	CCATGAACTTTCTGCTGTCTTG	TGCTCTACCTCCACCATGCCAAG
